# Impact of adoption of drought-tolerant maize varieties on total maize production in south Eastern Zimbabwe

**DOI:** 10.1080/17565529.2017.1372269

**Published:** 2017-09-07

**Authors:** Rodney Witman Lunduka, Kumbirai Ivyne Mateva, Cosmos Magorokosho, Pepukai Manjeru

**Affiliations:** a International Maize and Wheat Improvement Centre (CIMMYT), Harare, Zimbabwe; b The International Crops Research Institute for the Semi-Arid Tropics (ICRISAT), Bulawayo, Zimbabwe; c Department of Agronomy, Midlands State University (MSU), Gweru, Zimbabwe

**Keywords:** drought, drought-tolerant maize, maize production, food security, eastern

## Abstract

Drought is a huge limiting factor in maize production, mainly in the rain-fed agriculture of sub-Saharan Africa. In response to this threat, drought-tolerant (DT) maize varieties have been developed with an aim to ensure maize production under mild drought conditions. We conducted a study to assess the impact of smallholder farmers’ adoption of DT maize varieties on total maize production. Data for the study came from a survey of 200 randomly sampled households in two districts of Chiredzi and Chipinge in southeastern Zimbabwe. The study found that 93% of the households were growing improved maize varieties and that 30% of the sampled households were growing DT maize varieties. Total maize yield was 436.5 kg/ha for a household that did not grow DT maize varieties and 680.5 kg/ha for households that grew DT maize varieties. We control for the endogeneity of the DT adoption variable, by using the control function approach to estimate total maize production in a Cobb–Douglas model. The results show that households that grew DT maize varieties had 617 kg/ha more maize than households that did not grow the DT maize varieties. Given that almost all farmers buy their seeds in the market, a change in varieties to DT maize seeds gives an extra income of US$240/ha or more than nine months of food at no additional cost. This has huge implications in curbing food insecurity and simultaneously saving huge amounts of resources at the household and national levels, which are used to buy extra food during the lean season.

## 1. Introduction

### 1.1. Preamble

Drought has been highlighted as one of the major causes of reduced maize production and food insecurity across the globe and particularly in sub-Saharan Africa (SSA), where agriculture production is largely rainfed (Shiferaw, Prasanna, Hellin, & Bänziger, 2011). Daryanto, Wang, and Jacinthe (2016) estimated that the occurrence of mid-season droughts, particularly at the vegetative and productive phases for maize, reduces yields by 39.3%. Although projected changes in precipitation during the maize growing season in SSA vary with location and region (Cairns et al., 2013; IPCC, 2007), overall temperatures are predicted to increase by 2.1–3.6°C by 2050 (Cairns et al., 2012). The predicted increase in temperature is likely to have huge implications for maize production and, subsequently, the food security and livelihoods of smallholder farming households (Lobell, Bänziger, Magorokosho, & Vivek, 2011). Adapting to such climatic changes is thus critical for ensuring national food security and economic stability. One such adaptation strategy has been the development of drought-tolerant (DT) maize varieties. Thus, since the late 1990s, DT maize varieties have been viewed as part of the solution to sustaining maize production mainly under smallholder farming conditions. Since then, the development of DT maize varieties has remained a major objective of breeding programmes and research institutes across the globe (Bänziger, Setimela, Hodson, & Vivek, 2006; Campos, Cooper, Habben, Edmeades, & Schussler, 2004).

A DT maize variety is defined as a variety that can produce approximately 30% of its potential yield (1–3 t ha^−1^) after suffering water stress for six weeks before and during flowering and grain-filling (Magorokosho, Vivek, & MacRobert, 2009). Under controlled experimental research, DT maize varieties not only exhibit drought tolerance but are also high yielding, more than most current commercial hybrids (CIMMYT, 2013). For example, on average, DT maize hybrids yield 40% more than commercial checks under drought conditions (Setimela et al., 2014). Therefore, DT maize offers some insurance over mid-season droughts and dry spells and the potential to ensure a substantial maize harvest under mild drought conditions. Furthermore, *ex-ante* economic analyses suggest that if widely adopted by smallholder farmers, DT maize can provide substantial financial benefits through increased grain harvests and reduced risk. Additionally, Kostandini, Mills, Omamo, and Wood (2009) claim that adoption of such varieties can generate between USD$362 and USD$590 million in cumulative beneﬁts to both producers and consumers in SSA.

Although the ex-ante assessment of adoption of DT maize varieties has predicted positive impacts regarding yield potential, food security, and household income (Kostandini et al., 2009; La Rovere et al., 2014), ex-post evidence on the adoption of the DT varieties is inadequate. Additionally, there are only a few studies that have assessed the adoption levels of DT maize varieties among smallholder farmers in SSA overall, including Fisher et al. (2015), Holden and Fisher (2015), and Kassie, Jaleta, Shiferaw, Mmbando, and Mekuria (2013). Thus, to the best of our knowledge, there are no ex-post impact assessment studies on the adoption of DT maize in SSA. Therefore, using primary household data from the Chipinge and Chiredzi Districts of Zimbabwe, this paper provides novel information and empirical evidence on the impacts of the adoption of improved agriculture technologies by assessing the impact of adopting DT maize varieties on total maize production of smallholder farmers.

Zimbabwe is a suitable case study for the impact of the DT maize varieties because as in most SSA countries, in Zimbabwe, agriculture is the largest economic sector, contributing directly and indirectly to the livelihoods of more than 75% of the population (Kapuya et al., 2010). Furthermore, maize is a staple crop accounting for 40–50% of the calories consumed by the majority of the SSA population (FAOSTAT, 2010). Additionally, as in many SSA countries, maize in Zimbabwe is largely grown under rain-fed agriculture; hence, it is susceptible to variations in climatic conditions. Therefore, lessons and experiences drawn from Zimbabwe can be adapted and applied to other countries in SSA for the effective promotion of DT varieties to ensure food security.

### 1.2. Climate change and food production in Zimbabwe

Africa is recognized as one of the most vulnerable regions in the world to climate change due to widespread poverty and limited coping capacity (Madzwamuse, 2010; UNFCCC, 2007). Zimbabwe is particularly vulnerable due to its heavy dependence on rain-fed agriculture and sensitive climate resources (Chaguta, 2010). Climate records demonstrate that Zimbabwe is already experiencing the effects of climate change, notably rainfall variability and extreme events (Brown et al., 2012). The rainfall patterns in Zimbabwe are erratic and mostly characterized by acute mid-season dry spells and droughts, which render agricultural production unreliable. Climate change probability estimates show that moderate, severe, and extreme droughts are highly likely to occur in January–March at least twice every 10 years (Brown et al., 2012). Smallholder farmers have also reported a change in the weather pattern. Rurinda et al. (2013) reported that more than 90% of farmers in eastern Zimbabwe have perceived that the climate has changed, with increased rainfall variability characterized mainly by the late onset of rainfall and prolonged mid-season dry spells. They observed that the number of rain days per season has decreased with time, whereas the mean total annual rainfall has not changed, thus indicating an increased number of dry spells within the rainy season. Fisher et al. (2015) reported that farmers in southern Africa indicated having experienced 1–3 droughts during the past 10 years, with Zimbabwean farmers reporting the largest number of recent droughts, on average.

The effects of these climate changes and variability are being observed in agricultural production and livelihoods mainly of the rural Zimbabwean smallholder farmers. Kindie et al. (2015) predicted that Zimbabwe, like many other countries in SSA, would experience the highest reduction in maize yield due to climate change by 2050. Mano and Nhemachena (2007) showed that agricultural production in Zimbabwe’s smallholder farming system is significantly constrained by climatic factors (high temperature and low rainfall). Using a Ricardian approach, Mano and Nhemachena show that an increase in temperature of 2.5°C would result in a decrease in net farm revenue of $400 million for all farms in Zimbabwe. Specific to maize production, impacts of climate change have already shown huge negative effects at both the household and national levels. Between 1993 and 2000, average annual maize production stood at 1.64 million tons before dropping to 1.08 million tons between 2001 and 2008 (Brown et al., 2012). Zimbabwean farmers have faced significant economic constraints due to the increasing shortage of foreign currency for imports such as inorganic fertilizers and rising interest rates that have made credit unaffordable (Moyo, 2011). In addition to high temperature and low rainfall, those factors are significantly responsible for the decline in crop production.

An overview of Zimbabwean smallholder farmer’s adaptation to changing climate indicates that farmers are already using some adaptation strategies such as dry and early planting, growing drought resistant crops, changing planting dates, and using irrigation (Mano & Nhemachena, 2007). In Chiredzi District, Brown et al. (2012) showed that farmers have been planning and implementing some strategies including improvements in water availability, optimizing crop mix during the rainy season, and planting DT crops. The demand for DT crops such as maize and sorghum is increasing in several countries, including Zimbabwe (Cavatassi, Lipper, & Narloch, 2011; Fisher & Snapp, 2014; Westengen & Brysting, 2014). Fisher et al. (2015) found that the adoption of the DT maize varieties by smallholder farmers in SSA is becoming extensive. The genetic gains of DT maize varieties have proven to be higher than those of non-DT maize in both experimental stations and farmers’ field trials. Setimela et al. (2012) reported that the best new DTMA hybrids out-yielded the farmers’ varieties by more than 35% and 50% under low- and high-yield conditions, respectively, when compared to the most widely grown commercially hybrid varieties available in southern Africa. However, empirical evidence of the impact of DT maize remains limited.

A review by Fisher et al. (2015) of 19 recent relevant empirical studies, published in scholarly journals, covering 14 SSA countries and over 16,000 farmers, that examined the response of African farmers to extreme weather events and their attempts to adapt to perceived long-term environmental changes addressed two main questions. First, do smallholder farmers in SSA perceive climate as variable or changing? Second, what adjustments in agricultural practice have African farmers used to adapt to climate variability and change? We add to this literature by assessing the impact of the adjustments/adaptation strategies in maize production. We have singled out the adoption of DT maize varieties as an adaption strategy to climate change and evaluate whether farmers who planted these maize varieties had significantly more maize produced than farmers who planted ordinary maize varieties in drought-prone areas of southeastern Zimbabwe.

## 2. Materials and methods

### 2.1. Data sources

**Figure 1.**
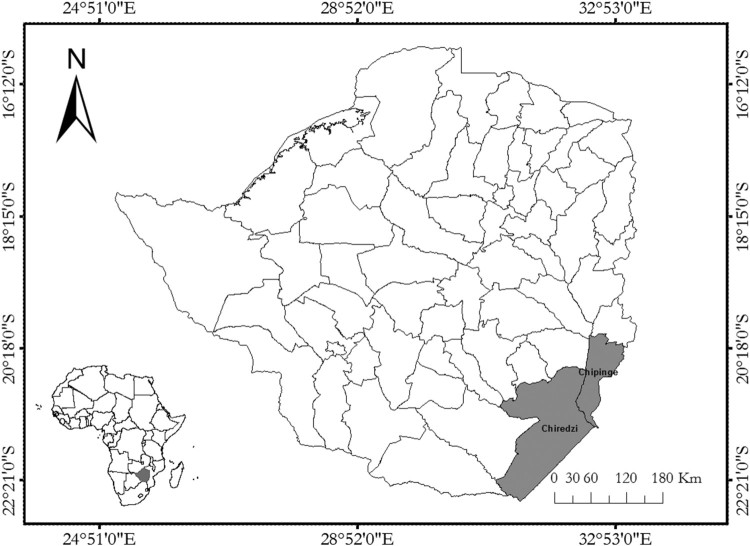
Map of Zimbabwe showing the study sites. The map was generated using ArcGIS 10.0.

The data used in this study come from a household survey that was conducted in the two districts of Chiredzi and Chipinge in southeastern Zimbabwe (Figure 1) during the months of April–June 2014. A total of 200 households were interviewed. The questionnaire was administered to the head of each household, but in their absence, the second most influential person in the household was interviewed. Before the start of the interviews, respondents were briefed on the purpose of the study and were informed that their participation in the study was voluntary. Furthermore, respondents were assured that their identity would not be disclosed to any third party. Information collected ranged from household demographics, socio-economic status, agricultural landholding, agricultural input use for maize production, maize varieties cultivated, and climate change awareness and response. The data were collected using a detailed questionnaire that was administered by seasoned enumerators and supervised by master’s students and the authors of the paper. The data collection was part of the monitoring exercise of the adoption of DT maize varieties by smallholder farmers under the Drought Tolerant Maize for Africa (DTMA) programme.

### 2.2. Sampled districts and sampling procedure

A multi-stage sampling technique was used to sample the 200 households in the two districts. The first stage was purposive where the two districts were selected for the study based on their drought proneness. Chiredzi has a total population of 275,759 people (64,134 households), while Chipinge has 298,841 people (64,675 households) (Zimstat, 2012). In the second stage, we randomly selected one ward (Ward 27) from Chiredzi and two wards from Chipinge (Wards 16 and 26). In each ward, study villages were randomly selected based on their population density. Therefore, five villages were randomly selected in Chiredzi, while two villages were randomly selected from Chipinge, i.e. one in Ward 16 and the other in Ward 26. This was done to give all households in the two districts equal probability of being sampled for the survey. A total of 67 households were randomly sampled from Ward 27 in Chiredzi district and 123 households from Chipinge district (67 households from Ward 16 and 66 from Ward 26).

### 2.3. Description of the sampled districts

Chiredzi is situated in Masvingo Province in the Southeast Lowveld at 21.03°S latitude and 31.57°E longitude at an average elevation of 392 metres above sea level (masl). The district is characterized as a medium drought-risk area, with annual average rainfall and annual average temperature of 450 mm and 19.4°C, respectively (Unganai, Murwira, Nherera, Troni, & Mukarakate, 2010). Downscaled empirical models for Zimbabwe show that Chiredzi District will have an increase in the annual surface temperatures of 1.5–3.5°C by 2046–2065 across the district (Brown et al., 2012). Chipinge District is situated in Manicaland Province in the Southeast Lowveld at 20.52°S latitude and 31.23°E longitude at an average elevation of 394 masl. Similar to Chiredzi, Chipinge is characterized as a medium drought-risk area; however, the annual mean rainfall is 600 mm which is higher than that of Chiredzi and a daily average temperature of 18.4°C. These figures tend to increase as one moves to the northern part of the district, which is mountainous and borders the highland of Chimanimani. The average annual rainfall in Chipinge North increases up to approximately 1105 millimetres per annum. Overall, these two districts present a good study area where farmers have experienced drought and are likely to have adopted different strategies that reduce the risk posed by drought, including planting DT maize varieties.

### 2.4. Description of the data used in the study

The study used two main dependent variables, i.e. (a) whether a household grew a DT maize variety in the 2013/14 growing season and (b) total maize harvested during the 2013/14 season. The first variable is a dummy where we estimated factors that determined whether the household decided to grow DT maize varieties. We first estimated the probability of the household growing a DT maize variety. The variable for whether a household grew DT maize variety was constructed from the names of varieties the farmers grew in the 2013/14 season. Household heads were asked to name the maize varieties they grew in the 2013/14 season. Using this list of maize varieties, we sorted expert information of the varieties from CIMMYT breeders and seed experts to group the varieties into DT or non-DT (Table 1).

**Table 1.** Maize varieties are grown by households in the sample and their traits.

Characteristic trait	Name of maize variety	Type of variety	% of plots
Drought tolerant	PAN 53	Hybrid	21.87
	SC 301	Hybrid	5.19
	PGS 61	Hybrid	1.95
	ZM 401	Open-pollinated variety	0.65
	ZM 521	Open-pollinated variety	0.22
Total % of plots			29.88
Non-drought tolerant	P2859W	Hybrid	25.54
	SC 513	Hybrid	11.69
	PAN 413	Hybrid	8.66
	SC 401	Hybrid	4.11
	R201	Hybrid	3.25
	PHB3253	Hybrid	1.73
	SC 403	Hybrid	0.87
	SC 635	Hybrid	0.87
	PHB30G19	Hybrid	0.65
	P2958W	Hybrid	0.65
	SC 501	Hybrid	0.43
	SC 533	Hybrid	0.43
	SC 633	Hybrid	0.22
	Hickory King	Local	3.46
	Kalahari	Local	0.22
	Red-cork	Local	3.03
Total % of plots			70.12

Variables on inputs used in the plot of maize were obtained from the responses to the household survey. Household heads were asked how much inorganic and organic fertilizer was applied on the maize plot. Using the values that households gave on the amount of fertilizer and the size of the plot they applied it on, we calculated the rate of fertilizer (organic and inorganic) that was applied to the maize plots. Household heads’ socio-economic information that could influence their decision to adopt the DT maize varieties was also included in the data analysis. This included age, gender, and educational qualification. Educational qualification was estimated by the number of years the household head spent in school.

### 2.5. Econometric analysis

To assess whether the adoption of DT maize varieties significantly affects the total maize production at the household level, we regress the total maize produced by the household with some input variables, with the DT maize adoption dummy variable as a production shifter. We test the significance of the DT maize adoption variable to check whether it is significant and positive, and we further assess the marginal changes on total maize produced by a household shifting from non-DT to DT maize varieties.

To begin with, we assumed a deterministic Cobb–Douglas production model given by the equation:

(1) $$\ln Q_{i}=f(\ln X_{ij}\beta_{j}+\text{D}{\text{T}}_{i}\delta |Z,\text{Cl}),$$

where $\ln Q_{i}$ is the natural logarithm of total maize harvested by household i using the natural logarithm of inputs ln⁡X from a set of different, but complementary inputs denoted by j. $\beta_{j}$ denotes the vectors of parameters to be estimated. $\text{D}{\text{T}}_{i}$ is the variable indicating whether household i grew DT maize, and δ is the estimated parameter. *Z* is the household characteristics, which can influence adoption of DT maize variety. These variables include the household head’s literacy level, the age of the household head, and the gender of the household head. These socio-economic variables have been found to influence the decision to adopt improved technology (Kassie et al., 2012) including for maize production and yield. Cl are climatic conditions (drought spells, and temperature). Climate variables such as temperature and rainfall were not available at the local level. However, district-level climate figures were available; they do not give enough variation between the households. Therefore, we use ‘household experiencing drought in the past five years’ as a proxy variable for *Cl*. Therefore, parameters are estimated from the equation, which is specified as:

(2) $$\ln Q=\beta_{0}+\ln\text{lan}{\text{d}}_{i}\beta_{1}+\ln\text{manur}{\text{e}}_{i}\beta_{2}+\ln\text{fer}{\text{t}}_{i}\beta_{3}+\ln\text{labou}{\text{r}}_{i}\beta_{4}+\text{DT}\beta_{5}+Z_{i}\beta_{6}+\text{Cl}\beta_{7}+\epsilon .$$

Given that factors that affect DT maize variety adoption also influence total maize production, the estimated parameters from Equation (2) will be inconsistent because of the endogeneity. Therefore, to control for this endogeneity of DT maize adoption, we estimate production of total maize production Q using the control function method. The endogenous variables will become appropriately exogenous in a second stage estimating equation by adding appropriate residuals to serve as the control functions. This method is appropriate for our analysis because our endogenous variable ‘DT maize adoption’ is a binary variable. Plugging in fitted values for the endogenous variable in the second stage or using two-stage least squares regression only works in the case where the first regression is linear. Due to the binary nature of the ‘DT adoption’ variable, this will lead to inconsistent estimation in the second stage. However, the control function approach fits this more appropriately. In addition, the control function approach makes it much easier to test the null that for endogeneity as well as compute average partial effects, which are main interest. The approach also leads to robust, regression-based Haussmann test for endogeneity of the suspected variables (Wooldridge, 2002). For the control function method, we first estimated the probability of DT adoption by smallholder farmers using a probit model.

(3) $$\text{DT}=1\;(\alpha_{0}+X_{j}\alpha_{\;j1}+Z_{i}\beta +u\geq 0),$$

where *DT* is the dummy variable equal to 1 if the household grew DT maize variety during the 2013/14 growing season and zero otherwise. *X* are *j* variables that affect the household decision to adopt DT maize varieties including experience of drought and heat stress in the past five years. u is the error term. In estimating the probability of adopting DT maize varieties, we also considered some factors that could influence farmers’ decision whether to adopt. We also include other household socio-economic variables (*Z*) such as the age of households head, the gender of household head, the number of years of experience in agriculture, the main income source for the household, and the education qualification of the household head as the total number of years the household head was in school. Using the predicted probability to adopt a DT maize variety, we calculate the error term and use in n the second production model, (see Equation (4))

(4) $$\ln\;Q=\beta_{0}+\ln\;\text{lan}{\text{d}}_{i}\beta_{1}+\ln\;\text{ma}{\text{n}}_{i}\beta_{2}+\ln\;\text{fer}{\text{t}}_{i}\beta_{3}+\ln\;\text{la}{\text{b}}_{i}\beta_{4}+\text{DT}\beta_{5}+\hat{u}\beta_{\mathrm{error}}+Z_{i}\beta_{6}+\text{Cl}\beta_{7}+\text{Lo}{\text{c}}_{i}\beta_{8}+\epsilon .$$

This is the Cobb–Douglas production equation with a control function. We take log transformations of the total maize production and the production factors, maize land size, organic manure, inorganic fertilizer, and total hired labour. However, we noticed that there are a number of zero values for input use for some households. This creates a problem since log⁡(0) is undefined. We deal with the problem by using the Inverse Hyperbolic Sine Transformation to take logs of the variables. We also add other factors that affect production at the household level and village dummies. We run an ordinary least squares model (OLS) with the estimated error term $\overset{\wedge }{u}$ from the probit model, as the control for the endogeneity of the DT adoption variable (*DT*) in the total maize production equation (control function approach) (Wooldridge, 2002). We are interested in the expected value of maize production with the change from non-DT maize to DT maize at the household level, and it is given by ∂E(ln⁡Q|DT)/∂DT. This is given by the coefficient $\beta_{5}$, and we estimate the marginal effects. A significant and positive result will indicate that growing DT maize varieties gives a household more maize than growing a non-DT maize variety. We will use this coefficient to calculate the value of the benefits using maize prices in Zimbabwe in the 2014 season. We control for other factors that affect maize production like pesticides, household characteristics (Z), and village location variables. The village dummies are used to control for variation as a result of the location that could affect area soil fertility and climate. This also controls for other unobserved location variables that affect maize production. We carried a variance inflation factor (VIF) tests to check for multicollinearity in our list of variables. All variables had a VIF below 10 and maximum is below 4. This gave confidence in the absence of multicollinearity from the regression.

## 3. Results and discussion

### 3.1. Descriptive analysis

Based on the sample in this study, ∼70% of the households grew non-DT maize varieties, while ∼30% of the households grew DT maize varieties. Table 1 shows that of the 30% that grew DT maize varieties, only 1% grew open-pollinated varieties (OPVs), i.e. ZM 401 and ZM 521. The majority of the households who grew DT maize varieties grew Pan 53 maize hybrid (∼22%). In general, most farmers (∼92% of the sampled households) grew hybrid maize, while only ∼7% grew local maize varieties. This is similar to some studies on improved maize seed adoption in Zimbabwe (Beyene & Kassie, 2015; Chikobvu, Kassie, & Lunduka, 2014). Chikobvu et al. (2014) found that 91% of the sampled households in six districts in Zimbabwe planted a hybrid maize variety during the 2011/12 growing season, while Beyene and Kassie (2015) reported that 95% of Zimbabwean smallholder farmers planted improved maize seeds.

**Figure 2.**
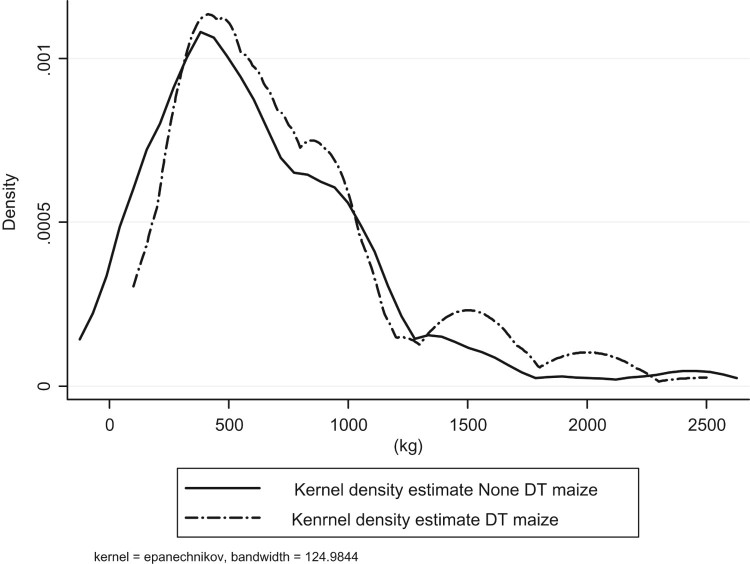
Kernel density estimates of DT and non-DT maize production at the household level.

Table 2 presents descriptive statistics of all the variables that are used in the study. On average, households that grew DT maize varieties during the 2013/14 growing season had significantly higher maize production than households that did not grow DT maize varieties. This study found that households that grew DT maize varieties had an average total maize production of 966 kg plot^–1^ with an average plot size of 3.55 acres, while households that did not grow DT had 716 kg plot^−1^ with an average land size of 4.1 acres. This translates to 272 kg acre^−1^ (680.5 kg ha^−1^) for a household that grew DT maize varieties and 174.6 kg acre^−1^ (436.5 kg ha^−1^) for households that did not grow DT maize varieties. This represents 56% more maize produced with the DT maize varieties than with non-DT maize varieties. This is higher than what has been reported in regional on-farm maize variety trials studies. Setimela et al. (2012, 2014) found that DT maize yielded 35–50% and 40% more than the best commercial hybrids, respectively. Our results compare not with the best commercial hybrids but with all other maize varieties that farmers grow, including some old varieties such as R201 and local varieties such as Hickory King. These should be low yielding; hence, our results show a higher increase in DT maize varieties than the regional on-farm varieties trials. The DT maize production graph is skewed to the right indicating dominance over non-DT maize varieties (Figure 2).

**Table 2.** Means, 95% confidence intervals, and *t*-statistics for variables used in the study.

Variable	Grew non-DT maize	Grew DT maize	*t*-Statistic
Land and maize production			
Average maize production (kg)	715.98	966.35	−3.26**
Total land endowment (ha)	3.77	3.86	−0.32
Total land allocated to maize (ha)	1.64	1.42	1.48
Inputs used			
Total organic fertilizer (manure) used on maize plot (kg/ha)	3.95	10.7	−1.75
Total chemical fertilizer used on maize plots (kg/ha)	1.64	0.93	0.55
Total pesticides used on maize plots (litres/ha)	0.37	0.75	−0.75
Dummy if households hire labour (1 = yes; 0 no)	0.15	0.18	−0.68
Total value of hired labour used on maize plots (USD$)	1.89	3.74	−1.29
Climate change perception of household			
Household has ever heard about climate change (%)	54.00	76.00	−4.15**
Household that experienced drought in last five years (%)	67.00	88.00	−4.16**
Household that experienced heat stress in last five years (%)	73.00	79.00	−1.23
Household social characteristics			
Average age of household (years)	46.46	44.70	1.05
Percentage of houses that were female headed (%)	47.00	42.00	0.91
Household head: number of years in school (years)	6.25	7.65	−3.53**
Household head: experience in agriculture (years)	20.66	17.52	1.66
Household size (ha)	6.19	6.52	−1.11
*N* (number of maize plots)	592	208	

*Significant at 0.10.

** Significant at 0.05.

*** Significant at 0.01%.

**** Significant at 0.001.

It is interesting to note that households that had heard about climate change (76%) and reported to having had experienced drought in the past five years (88%) were more likely to grow DT maize varieties than households that reported not to have had experienced drought in the past five years (Table 2). The level of education also seemed to have an influence on the decision to adopt DT maize varieties. However, input use such as organic fertilizer, chemical fertilizers, and pesticides seems to have had less influence on the decision to adopt DT maize varieties.

### 3.2. Probability of adopting DT maize varieties

We first present the probability model results of households growing a DT maize variety during the 2013/14 growing season. The results in Table 3 highlight that being aware of climate change and having had experienced drought in the past five years greatly influence the decision to adopt DT maize varieties. Having had experienced drought in past five years increased the probability of adopting DT maize varieties by ∼97%. Etwire, Al-Hassan, Kuwornu, and Osei-Owusu (2013) also found that in Ghana, noticing unpredictable temperatures was one of the factors influencing farmers’ adoption of climate-related strategies. As droughts are more frequent in Zimbabwe, DT maize varieties may be popular adaptation technologies that can be used by smallholder farmers. Fisher et al. (2015) found that most farmers in southern Africa have experienced 1–3 drought years in the past 10 years. However, Zimbabwean farmers, in particular, reported 4–5 years of drought in the past 10 years (Fisher et al., 2015). Considering the high experience of drought in Zimbabwe, the low level of adoption may indicate the presence of other underlying factors limiting the adoption of DT maize varieties. We discuss these in the next section.

**Table 3.** Probability model, estimating determinants of adoption of DT maize varieties.

Variables	Coefficients	Robust standard errors
Household has heard about climate change (1 = yes; 0 = no)	0.292**	(0.12)
Household experienced drought in last five years	0.973****	(0.16)
Household experienced heat stress in last five years	−0.001	(0.13)
Age of household head	0.000	(0.01)
Gender of household head (1 = female; 0 = male)	−0.194*	(0.11)
Household head: years of education (years)	0.085****	(0.02)
Household head: experience in agriculture (years)	0.004	(0.01)
Household size	0.097****	(0.03)
Agriculture is the main livelihood.	0.009	(0.05)
Total land endowment of household (acres)	−0.040*	(0.02)
Total land planted to maize (acres)	0.002***	(0.00)
Total fertilizer used by household (kg)	−0.001	(0.01)
Total amount of pesticides used by household	−0.002	(0.02)
Total hired labour by household	0.253	(0.20)
Village dummies		
Village 2	−0.527****	(0.15)
Village 3	−0.993****	(0.28)
Village 4	0.512**	(0.21)
Village 5	0.864****	(0.21)
Village 6	−0.482**	(0.24)
Village 7	0.050	(0.21)
Constant	−2.694****	(0.38)
Prob > chi^2^	0.000	
Number of observation (N)	788.000	
Pseudo *R*-squared	0.1757	

* Significant at 0.10.

** Significant at 0.05.

*** Significant at 0.01%.

**** Significant at 0.001.

Other variables show that household head’s literacy level, household size, households’ total land endowment and total cultivated land under maize were important factors behind the adoption of DT maize varieties (Table 3). The household head’s level of education was higher for DT maize adopters compared with non-adopters. This is expected, as educated households are more innovative and are risk takers when considering technology adoption. Foster and Rosenzweig (2010) indicated that educated individuals process information about new technologies more quickly and effectively than uneducated individuals. An increase in the household size increased the probability of adopting DT maize varieties by ∼9%. This could be attributed to the fact that as the household size increases, demand for food also increases. Therefore, to ensure a sustainable and secure food source, households will tend to favour less risky and higher yielding varieties.

Interestingly, total land endowments were negative and significantly correlated with the probability of growing DT maize varieties. While households with large land endowments can increase their yields and production through extensive farming or spatial expansions, households with small land endowments aim at maximizing their production and maize yield through intensification methods including the use of improved high-yielding varieties. Furthermore, households with smaller land endowments tend to be cautious and risk-averse; hence, growing maize varieties that have higher chances of escaping drought is a more favourable option for them. In addition, Kassie et al. (2012) indicated that the proportion of land allocated to any crop is an important indicator of the significance of the enterprise. Since maize is a very important food crop in the study area, as total land endowment decreases, the proportion of land allocated to maize increases. In addition, households with larger land endowments have the opportunity to grow other food and cash crops to diversify their harvest in drought years.

In terms of input use, none of the input variables, e.g. chemical fertilizers, labour, and pesticides, significantly affected the decision to adopt DT maize varieties. This is plausible because DT maize varieties do not have any extra or different input requirements from any other maize varieties. The model results also showed that households in Chiredzi are more likely to adopt DT maize varieties than households in Chipinge. Chiredzi is drier and has a lower average rainfall than Chipinge; thus, DT maize varieties may be more appealing to farmers, as they promise insurance over the drought.

### 3.3. Reasons for not growing DT maize varieties

**Figure 3.**
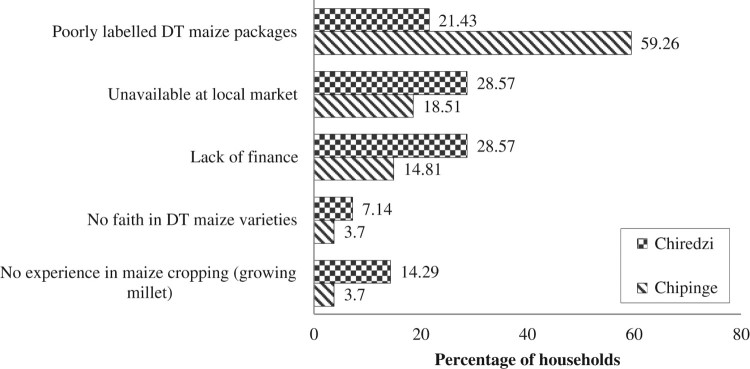
Reasons for not growing DT maize varieties in 2013.

Households that did not grow DT maize varieties were asked for the reasons behind their decisions. The households highlighted a number of important policy-related issues. In Chiredzi, households that did not grow DT maize varieties attributed this to ‘lack of finance’ (28.57%) to purchase the maize seeds on the market, ‘poorly labelled DT maize packages’ (21.43%), and ‘unavailability of DT maize at local market’ (28.57%) as their three most relevant reasons (Figure 3). In Chipinge, farmers reported ‘poorly labelled DT maize packages’ (59.26%), ‘unavailability of DT maize at local market’ (18.51%) and the lack of finance (14.81%) as their three most relevant reasons (Figure 3). Similar findings have been reported in Ghana by Tambo and Abdoulaye (2012) where farmers identified the cost of DT maize varieties and complementary inputs as constraints to their adoption. Fisher et al. (2015) also found that major barriers to adoption of DT maize in eastern and southern Africa include the unavailability of improved seed, inadequate information and resources, high seed price, and farmers’ perceptions of variety attributes. These factors require policy intervention in order to bring the seeds closer to the farmers at lower prices. Input subsidies can increase adoption of DT maize varieties, for example, the Farm Input Subsidy Program (FISP) implemented in Malawi since 2005/06 (Lunduka, Ricker-Gilbert, & Fisher, 2013). It assists ∼50% of farmers in Malawi to receive subsidized fertilizer for maize production, with additional vouchers for tobacco fertilizers and free modern maize seed (Lunduka, Ricker-Gilbert, & Fisher, 2013). Additionally, Holden and Fisher (2015) found that between 69% and 82% of sampled farmers who received an FISP voucher for maize seed redeemed their coupon for a DT maize variety. This may explain why Malawi is performing relatively well in disseminating DT maize to farmers (Fisher et al., 2015).

### 3.4. The impact of DT maize variety on maize production

Table 4 presents the estimated coefficients of the Cobb–Douglas production model with bootstrap standard errors clustered at the household level. Two models were estimated, first with maize production inputs only and the second with other production shifters including the adoption of DT maize varieties. In both models, the Chi-square and the *F* statistics were significant, indicating that the model was well specified. The results of Model A show that total maize area and hired labour are significant and positively affecting maize production. Model B presents results from the Cobb–Douglas production model with production shifters and our variable of interest, ‘grew DT maize varieties’, with the error term from the probit model $\overset{\wedge }{u}$ as a control function. The model results show that the variable ‘grew DT maize variety’ is positive and significant, indicating that total maize produced by a household was higher when the household grew DT maize varieties than non-DT maize varieties. The error term $\overset{\wedge }{u}$ in the model ‘B’ with control function is statistically significant, indicating the endogeneity of the variable ‘grew DT maize variety’ in the model.

**Table 4.** Regression results of total maize production.

Variables	CDonly (*A*)		Control function (*B*)	
	Coefficients	Robust standard errors	Coefficients	Bootstrap Standard Errors (400 reps)
Log of total fertilizer used	−0.043	(0.07)	−0.045	(0.06)
Log of total manure used	0.032	(0.03)	0.017	(0.04)
Log of total maize areas	0.644****	(0.08)	0.423**	(0.19)
Long of total hired labour	0.169****	(0.04)	0.159***	(0.05)
Planted DT maize (1 = yes; 0 = otherwise)			3.288**	(1.48)
Predicted error term ($\overset{\wedge }{u}$)			−3.027**	(1.44)
Household ever heard of climate change (1 = yes; 0 = otherwise)			−0.098	(0.16)
Household experienced drought in last 5 years (1 = yes; 0 = otherwise)			−0.495	(0.33)
Household experienced heat in last 5 years (1 = yes; 0 = otherwise)			0.510***	(0.19)
Gender of household head (1 = female; 0 = otherwise)			0.022	(0.20)
Age of households head (years)			0.005	(0.01)
Household size (*N*)			−0.063	(0.06)
Household head years of education (years)			−0.048	(0.05)
Household head year of agricultural experience			−0.011	(0.01)
Agriculture is the main livelihood			0.095	(0.06)
Total amount of pesticides used by household			0.004	(0.02)
Village dummies				
Village 2			0.902****	(0.27)
Village 3			0.672*	(0.40)
Village 4			−0.673*	(0.37)
Village 5			−0.983	(0.74)
Village 6			0.930***	(0.29)
Village 7			−0.107	(0.49)
Constant	5.391****	(0.12)	5.040****	(0.71)
Wald chi^2^(22)			271.05	
*F*(4, 795)	23.11			
*F*(22, 765)			12.76	
Prob > chi^2^	0.000		0.000	
Number of observations (*N*)	800.000		800.000	
*R*-squared	0.136		0.2934	
Adjusted *R*-squared			0.2734	

* Significant at 0.10.

** Significant at 0.05.

*** Significant at 0.01%.

**** Significant at 0.001.

Estimating the marginal effect of growing DT maize varieties at the predicted mean value of maize production of 687 kg shows that a change from non-DT to DT maize varieties increases total maize production by 876.71319 kg/3.55 acre plot ∼617 kg/ha (247 kg acre^−1^). This translates to USD$95 acre^−1^ (USD$240 ha^−1^) in additional income assuming that the price of maize is at USD$ 0.39 kg^−1^ grain, (2014 maize price in Zimbabwe). In addition to showing the superiority of the DT maize varieties, the results of this analysis show that the impact of DT maize varieties could have more benefits than changing other limiting resources. For example, increasing maize land size by an extra acre increases total maize production by 42 kg, while changing variety from non-DT to DT maize variety provides an increase of 247 kg acre^−1^. Such an increase in maize production is associated with no increase in the production cost since households already purchase maize seed on the market. In addition, we have no reason to believe that the associated costs of producing DT maize are different from non-DT maize. Therefore, promotion and support to DT maize varieties should be continued and scaled out to ensure increased maize production not only in drought-prone areas but also in areas that experience dry spells during normal maize growing seasons.

However, these results are conservative because some households that grow both DT and non-DT maize varieties may have a reduced total maize production. To estimate such results, there is need to control for unobserved heterogeneity both at the household and at the plot level using a panel data set. More detailed plot-level production data could also provide more insights on the benefit. However, we believe that these results are robust enough. Inasmuch as the control function controls for the endogeneity of the DT adoption, a panel data set could also control for unobserved heterogeneity of the farmers.

## 4. Conclusions

This study evaluates the impact of adoption of DT maize varieties on the total maize production of households in southeastern Zimbabwe. Using a data set of 200 randomly selected households, the study employs a control function model to estimate the impact of DT maize varieties on total maize production. The study finds that households that grew DT maize varieties had a very significant increase in the total maize production. Households that grew DT maize had 247 kg acre^−1^ more maize than those households that did not grow DT maize varieties. This translates into USD$240 ha^−1^ extra income for those households that grow DT maize varieties. These findings are similar to on-station and on-farm trials that have shown that DT maize is better yielding than the currently popular non-DT maize varieties.

In Zimbabwe, the production and productivity of maize have been decreasing since the early 1990s. From a surplus producer of maize, Zimbabwe has become a net food importer during the past decade (GoZ, 2012). Climate change is contributing significantly to this decline. Promotion of DT maize varieties in the country will help to contribute to the increase in total maize production and yield. The current Zimbabwe Agricultural Policy strategy does not clearly address climate change and how farmers should be assisted to adopt to the adverse effects. DT maize varieties are consistently showing potential to increase maize production both on station research (Setimela et al., 2017) and on farmers’ field as shown in this study. Farmers should be encouraged and informed of these new varieties with the DT trait. Reasons for not growing the DT maize varieties reported and documented during this study require that seed company properly label their seed packages to convey the proper message to the farmers. This reinforces the need for appropriate branding or labelling of DT variety attributes by seed companies. In addition, increasing the production of the DT maize seeds and making them available on the market is critical to ensure that farmers can easily find them in the markets. These policies require that they be well stipulated in the agricultural policy and strategies.

## Disclosure statement

No potential conflict of interest was reported by the authors.

## Supplemental data

Supplemental data for this article can be accessed at doi:10.1080/17565529.2017.1372269.

## Supplementary Material

Additional_materials_VIFs.docx
