# Supplementary material for: Impact of adoption of drought-tolerant maize varieties on total maize production in south Eastern Zimbabwe
Source: Clim Dev. 2017 Sep 7;11(1):35–46. doi: 10.1080/17565529.2017.1372269 (PMC6397629; doi:10.1080/17565529.2017.1372269)
Supplement: Additional_materials_VIFs.docx [file TCLD_A_1372269_SM9590.docx]

Additional materials

# Variance inflation factor (VIF) for the variables used in the regression.

After running the regression analysis, we tested for the presence of multicollinearity in the model. We found that all variables had VIF below 10 and highest VIF was 3.69 for “Age of the household head”. This gave use confidence that there was worry of multicollinearity in the regression.

| Variable | VIF | 1/VIF |
| --- | --- | --- |
| Age of household head | 3.69 | 0.271203 |
| Household head experience in agriculture | 3.32 | 0.301325 |
| Household size | 2.50 | 0.400305 |
| Agriculture main source of income | 2.41 | 0.415750 |
| Village 2 | 1.67 | 0.599695 |
| Household head literacy level | 1.58 | 0.633002 |
| Log of maize land size | 1.35 | 0.741771 |
| Household experience heat wave in last five years | 1.31 | 0.763700 |
| Village 7 | 1.27 | 0.786570 |
| Household experience drought in past five years | 1.25 | 0.802037 |
| Village 4 | 1.25 | 0.802937 |
| Village 5 | 1.24 | 0.809695 |
| Gender of household head | 1.22 | 0.816522 |
| Village 3 | 1.22 | 0.817940 |
| Log of inorganic fertilizer used | 1.19 | 0.837064 |
| Village 6 | 1.19 | 0.840852 |
| Total pesticides used | 1.19 | 0.841467 |
| Household head has heard about climate change | 1.18 | 0.845674 |
| Log of hired labour | 1.11 | 0.896865 |
| Log of organic manure applied | 1.11 | 0.899957 |
|  |  |  |
| Mean VIF | 1.61 |  |
